# Macro and Micro Routes to High Performance Bioplastics: Bioplastic Biodegradability and Mechanical and Barrier Properties

**DOI:** 10.3390/polym13132155

**Published:** 2021-06-30

**Authors:** Olivia A. Attallah, Marija Mojicevic, Eduardo Lanzagorta Garcia, Muhammad Azeem, Yuanyuan Chen, Shumayl Asmawi, Margaret Brenan Fournet

**Affiliations:** 1 Materials Research Institute, Athlone Institute of Technology, N37 HD68 Athlone, Ireland; oadly@ait.ie (O.A.A.); e.lgarcia@research.ait.ie (E.L.G.); m.azeem@research.ait.ie (M.A.); yuanyuanchen@ait.ie (Y.C.); mfournet@ait.ie (M.B.F.); 2 Fundamental and Applied Science Department, Universiti Teknologi PETRONAS, Bandar Seri Iskandar 32610, Perak Darul Ridzuan, Malaysia; muhammad_24531@utp.edu.my

**Keywords:** biomaterials, biodegradation, bioplastics, mechanical performance, barrier performance, processability

## Abstract

On a score sheet for plastics, bioplastics have a medium score for combined mechanical performance and a high score for biodegradability with respect to counterpart petroleum-based plastics. Analysis quickly confirms that endeavours to increase the mechanical performance score for bioplastics would be far more achievable than delivering adequate biodegradability for the recalcitrant plastics, while preserving their impressive mechanical performances. Key architectural features of both bioplastics and petroleum-based plastics, namely, molecular weight (*M_w_*) and crystallinity, which underpin mechanical performance, typically have an inversely dependent relationship with biodegradability. In the case of bioplastics, both macro and micro strategies with dual positive correlation on mechanical and biodegradability performance, are available to address this dilemma. Regarding the macro approach, processing using selected fillers, plasticisers and compatibilisers have been shown to enhance both targeted mechanical properties and biodegradability within bioplastics. Whereas, regarding the micro approach, a whole host of bio and chemical synthetic routes are uniquely available, to produce improved bioplastics. In this review, the main characteristics of bioplastics in terms of mechanical and barrier performances, as well as biodegradability, have been assessed—identifying both macro and micro routes promoting favourable bioplastics’ production, processability and performance.

## 1. Introduction

Pervasive plastics are leaving an indelible imprint on our planet. As high performance and energy-saving materials, plastics are ubiquitous and central to socio-economic advancement. Current mainstay plastics are processed from fossil fuel resources, with production requirements expected to double over the next 20 years. After use, these recalcitrant plastics are contributing to waste stockpiles and alarming pollution. Recycling technologies, which primarily include mechanical and thermochemical approaches, does not meet the efficiency levels required to safeguard the planet and adequately revalorise plastics as new products. The current linear economic model of resource mining, use and discarding, is now widely recognised as unsustainable. A circular approach, where resources are repurposed cyclically, akin to biological lifecycles, is essential in achieving a sustainable socio-economic ecosystem.

Nature readily operates elegant and efficient regenerative cycles for natural polymers and end of life bio-based materials. Such biodegradation and bio-regeneration processes involve microbial, enzymatic and biocatalytic activities for depolymerisation and repolymerisation. Petroleum-based plastics, with their smooth surface topographies, extensive hydrophobic chains and lack of bio-accessible organic chemical groups, are strongly bio-inert and largely incompatible with bioprocessing, leading to their persistence over century timescales within land and water environments. Biomass provides a wealth of renewable and bio-waste resources for bioplastics synthesis. Many of these bio-based plastics, encompass capacities for biodegradation and bioprocessing with high performance features akin to petroleum-based plastics. The realisation of bioplastics that exhibit a complete set of mechanical and biodegradability, hold the promise of delivering material of ecologically sustainable, low carbon footprint circularity.

Bioplastics to date, however, have not achieved wide acceptability by the industry. Incompatibility with existing sorting infrastructures and high temperature mechanical recycling implemented for fossil-based plastics, along with raised production costs, are limiting factors. Technical shortcomings, such as brittleness, lower gas barrier functions and processing performances, have also played a role in keeping current market penetration levels in the region at just 2%. Combining high performance for consumer applications and continuous low carbon closed loop regeneration within plastics poses considerable challenges. At a fundamental structural level, polymeric features associated with good mechanical and fluid barrier properties are typically prohibitive to biodegradability. Petroleum-based plastics achieve the required degrees of high mechanical strength combined with flexibility and strong liquid and gas barrier properties by packing their sleek chemically structured chains into signature crystalline and amorphous regional arrangements. The tight alignment of chemically simple chains at high degrees of crystallinity also renders these plastics largely incompatible with biodegradation processes that require bioactivities, including enzymatic hydrolysis. Bioplastics, in contrast, by the very fact that they are generated from bio-based resources, are inherently more complex with more elaborate chemical structures. This provides both a means to progress their mechanical performance properties and provides amenability to bioactivity with higher levels of hydrolysable groups available for post use biodegradation and biodepolymerisation. To date, equivalent results are readily achievable, and in cases, results outperform particular mechanical properties for polylactic acid (PLA) and polyhydroxyalkanoates (PHA) bioplastics compared with conventional fossil-based thermoplastics. The potential to address performance limitations by a combination of bottom up and top-down approaches using considered chemical structure modifications and blending and composite formations, holds the promise of framing a new generation of bioplastics that encompass sustainability with performance.

In this review, the sustainability/performance triangulation between the biodegradability, mechanical and barrier properties of bioplastics is discussed. Approaches to overcoming the gap between industrially required mechanical and barrier performances and biodegradability are overviewed and related to the potential to build a new generation of high-performance sustainable plastics.

## 2. Bioplastic Production

Biopolymers can be obtained directly from biomass, as in the case of proteins and polysaccharides and synthetic biopolymers, such as PLA. Biodegradable polymers, including polycaprolactone (PCL), polyglycolic acid (PGA) and polybutylene succinate-co-adipate (PBSA), are primarily synthesised from petrochemicals. Microbial fermentation of biopolymers, including PHA and bacterial cellulose (BC), operates under relatively benign low energy conditions, and hence, is a highly favourable sustainable production route. Various microorganisms can accumulate PHAs as storage materials when cultivated under different nutrient and environmental conditions. This ability allows their survival under stressful conditions. The number and size of the PHA granules, the monomer composition, macromolecular structure and physico-chemical properties vary, depending on the producing microorganisms, the feedstock supplied and the operation conditions [1,2,3]. On the other hand, some bacteria can produce BC. This exopolysaccharide is a naturally occurring, chemically pure, free of hemicellulose, lignin and pectin, which is why BC purification is an easy process demanding low energy consumption. Nevertheless, production yields are very low, cultivation times are extensive, and thickness of layers is limited, which are major drawbacks in the conventional BC production process, affecting the range of possible applications [4]. For the industrial production of bioplastics, three limitations are very important. These include requirements of specialised growth conditions, expensive precursors, and high recovery costs. Building an increased body of knowledge on producing microbes’ metabolism, biosynthetic pathways and their regulation is essential in overcoming these limitations [1,5].

## 3. Biosynthesised Plastics

In recent years, PHA polymers have emerged as one of the most promising biodegradable materials. Unlike commonly used fossil-based plastics or PLA, which requires the additional step of lactic acid polymerisation, PHAs are the product of bacterial metabolism and have a function of cytoplasmic inclusions. These materials, thanks to the over 150 monomer units, can have a variety of polymer properties that can compete with commonly used plastics, such as polyethene or polypropylene [6]. Monomer composition is connected to the substrate specificity of PHA synthase, hence the type of derived PHA is highly connected to the microorganism used for its production. For example, biosynthesis of short-chain length PHAs (SCL-PHAs) consisting of poly-3-hydroxybutyrate P(3HB) homopolymers is a three-step process regulated by 3-ketothiolase, acetoacetyl-CoA reductase and the SCL PHA synthase [7,8]. Bacteria, such as *Aeromonas caviae* and *Pseudomonas stutzeri*, are proven producers of PHA synthases with wide substrate specificity. Moreover, these enzymes have been identified after recombinant expression in *Ralstonia eutropha*, previously PHA negative. These PHA synthases can produce copolymers of SCL- and medium-chain length PHAs (MCL-PHA) [9]. It has been reported that combinatorial mutations in *P. aeruginosa*, *P. oleovorans*, and *P. putida* resulted in their ability to synthesise PHAs with 3-hydroxybutyrate (3HB), 3-hydroxyhexanoate (3HHx), 3-hydroxyocatanoate (3HO), 3-hydroxydecanoate (3HD), or 3-hydroxydodecanoate (3HDD) monomers [10,11,12,13]. These structures are presented in Table 1. The development of recombinant strains and using genetic engineering techniques can lead to improved mechanical and thermal characteristics of PHA materials. These properties are highly dependent on various factors during upstream and downstream processes. Modification of PHAs chemical structure, such as the introduction of functional groups or producing blends and copolymers, can also affect the quality of these materials [14]. Traditional chemical synthesis techniques have been successfully used for creating block copolymers with PHA materials. For example, block copolymers, including PHB blocks balancing with other materials (poly(6-hydroxyhexanoate), poly(3-hydroxyoctanoate), monomethoxy-terminated poly(ethylene glycol) (mPEG), and poly(ethylene glycol) (PEG)), have been reported. These structures are presented in Table 2 [8,15].

PHAs can also be derived from various substrates, including industrial waste streams [16], food waste [17], supplemented solid biodiesel waste, plant oils [7]. Besides these, seaweeds were found to be great feedstock for PHAs productions [18]. These materials can be produced using different strategies, including batch, fed-batch and continuous processes, and various conditions could be used for their conduction. Batch cultivations are easy and simple to operate, but production yields are very low. On the other hand, fed-batch cultivation can provide higher product and cell concentrations (no substrate inhibition) [19]. Continuous cultivation are also considered to be a viable strategy, providing required concentrations of limiting substrates, such as fatty acids and their derivatives [20]. Nevertheless, large scale application is still impractical with this type of production. Another strategy used for PHA production is solid state fermentation (SSF)—microbial cultivation on solid support made of appropriate substrates. SSF can be performed with inexpensive cultivation media, such as substrates, based on agro-industrial residues. This strategy provides disposing of waste, while valuable compounds are being produced at the same time [21]. There are few additional advantages of SSF over submerged fermentation: Easier aeration, higher substrate concentration, as well as reduced downstream processing steps. However, keeping conditions constant during the process is the biggest drawback to the market-ready production of PHAs using this strategy [22].

Bacteria from the genus *Komagataeibacter* (former *Gluconacetobacter*) synthesises another interesting, biosynthesised bioplastic—bacterial cellulose (BC). The BC production is a multi-step, strictly regulated process that involves regulatory proteins, few enzymes and catalytic complexes. The formation of 1,4-β-glucan chains is the first step in BC synthesis. This process, together with chains’ assembly and crystallisation, occurs intracellularly. The second step involves extracting cellulose chains from the cells and their assembly in fibrils [4,23]. The yield and properties of the resulting material highly depend on the used bacterial strain and conditions of the conducted process, including medium composition, aeration, shaking, etc. Morphological and physical properties of the resulting material are in correlation to the cultivation broth composition. BC production can be improved using genetically modified producing strains, isolating novel strains with the ability to produce BC, and investigating process parameters and their significance. Florea et al. isolated *Komagataeibacter rhaeticus* strain able to produce a high yield of BC, while growing in low nitrogen level medium [24]. In order to increase BC production in limited oxygen conditions, genetically engineered strains have been developed [25]. Hungund et al. reported using ethyl methanesulfonate and ultraviolet radiation for *Gluconacetobacter xylinus* NCIM 2526 strain improvement that resulted in a significantly higher yield of BC (30%) [26].

**Figure 1:**
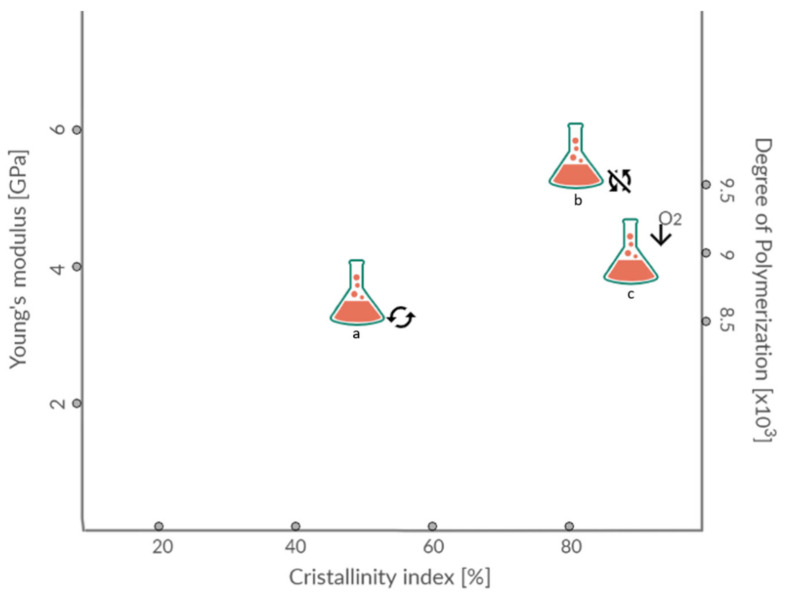
Young’s modulus, crystallinity index and degree of polymerisation of BC depending on cultivation conditions: a—with shaking, b—without shaking, c—with additional oxygen supply.

Different strategies can be used to produce BC, including aerated submerged cultivation, static culture and airlift bioreactors [27]. Effects of these conditions on cellulose mechanical properties are presented in Figure 1.

Tensile strength, polymerisation degree and crystallinity index are highly influenced by BC structure. Produced in static conditions, BC forms pellicles on the surface of the medium. This gelatinous membrane can vary in thickness up to few centimetres depending on substrates’ availability. Produced like this, BC has a significantly higher crystallinity index and tensile strength than the BC produced in agitated culture. Supply of air in agitated cultures will result in pellets with a higher crystallinity index. Crucial factors in BC production are the design of the reactor and proper control of conditions and process. These parameters highly affect the yield and quality of the resulting material. No matter which strategy is used, pH, oxygen supply, and temperature are essential conditions that should be carefully monitored. These parameters are mostly strain-dependent, but it was shown that the optimal pH value for cell growth and BC production is usually between 4.0 and 7.023, while the optimal temperature is in the range 28–30 °C [28].

Despite the great mechanical properties of the resulting material, static culture cannot provide uniformity of the cultivation broth; hence, cells are not equally exposed to nutrients and the thickness of the BC layer can be uneven. Additionally, productivity achieved in this strategy is very low, and it demands an extensive period of cultivation [29,30]. To improve the productivity of this process, the fed-batch strategy was developed, and a constant BC production rate was achieved for 30 days [31]. Submerged cultivations with agitation provide uniformity of nutrients, especially oxygen, resulting in higher yields in comparison to the static cultivations and making the production process cost-effective. Products of agitated fermentation depend on applied agitation speed and may include various forms of cellulose: Spheres, pellets, fibrous suspension [32]. As previously mentioned, higher productivity is the biggest advantage of submerged compared to static cultivation, but drawbacks, such as products’ shape consistency and limited mechanical properties, are issued to overcome [33]. Another problem that can limit BC yield in aerated cultivation is a synthesis of gluconic acid. Due to the high agitation rates and hydrostatic stresses, the production of the secondary, protective metabolites is favoured over cellulose. Nevertheless, submerged cultivation of BC was implemented in different types of bioreactors, such as airlift, stirred tank and rotating disk. Production of this material on a large scale is still an issue, and designing new or improving existing equipment for this purpose is an important subject of research [34].

### 3.1. Bioplastic Mechanical Performance

**Figure 2:**
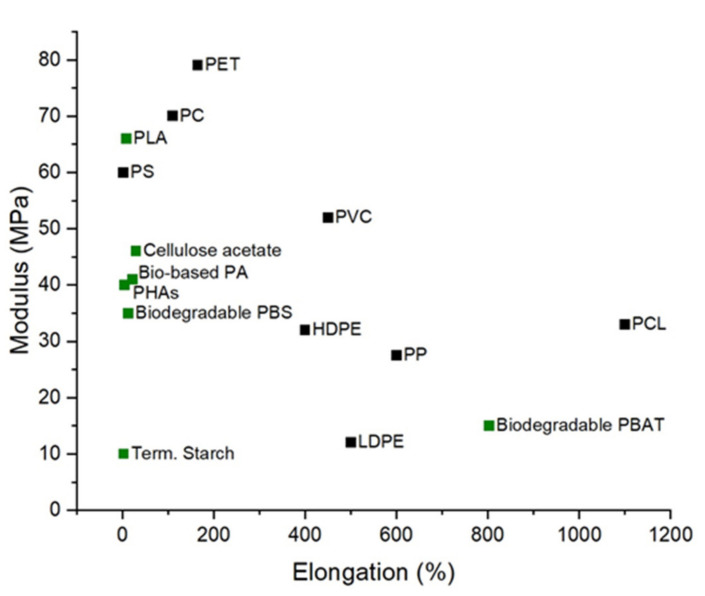
Maximum Tensile Strength (MPa) and Maximum Tensile Elongation (%) of bioplastics compared to petroleum-derived plastics, Data from Ref. [36].

Bio-based polyesters (PLA, and PHAs) exhibit similar mechanical properties and can even exceed conventional plastic performances. Figure 2 demonstrates the Maximum Tensile Strength (MPa) and maximum Tensile Elongation (%) of bioplastics compared to petroleum-derived plastics. PLA is one of the most prominent bioplastics in terms of global consumption. It possesses several desirable properties, such as biocompatibility, biodegradability, composability and low toxicity to humans. The mechanical properties of PLA are greatly affected by the degree of PLA crystallinity. PLA derived from 93%, or more L-lactic acid can be semi-crystalline, while it is strictly amorphous when derived from 50–93% L-lactic acid. Thus, high tensile strength can be observed in films of high L-lactide content. Tensile strength and impact resistance are also influenced by the degree of crosslinking and the annealing of L-PLA, which increases the stereoregularity of the chain [35]. Comparison of mechanical properties between poly(98% L-lactide) and poly(94% L-lactide) showed a slightly greater elongation at yield for 98% than 94% L-lactide. However, poly(94% L-lactide) has an elongation at the break seven times greater than poly(98% L-lactide), indicating more plastic behaviour with 94% of L-lactide [35]. 

For racemic mixtures, a study by Chen et al. demonstrated that polymerisation of 50% D-Lactide and 50% L-Lactide usually results in forming an amorphous polymer of poly (DL-lactide) [37].

As a packaging material, PLA offers high stiffness (greater than polyethene terephtalate (PET) and polystyrene (PS)), good clarity (similar to PET), relatively low processing temperatures, excellent resistance to fats and grease, and good breathability suitable for fruits and vegetable storage. Such characteristics make PLA a potential candidate to replace PS, polyethene (PE) and polypropylene (PP) in the fabrication of disposable cups, salad boxes and cold food packaging [38]. Nevertheless, PLA brittleness with less than 10% elongation at the break renders it unsuitable as a pure material for applications that require plastic deformations at higher stress levels [39]. Additionally, PLA’s poor gas moisture permeability performance make it unsuitable for many beverage bottle applications [38].

**Figure 3:**
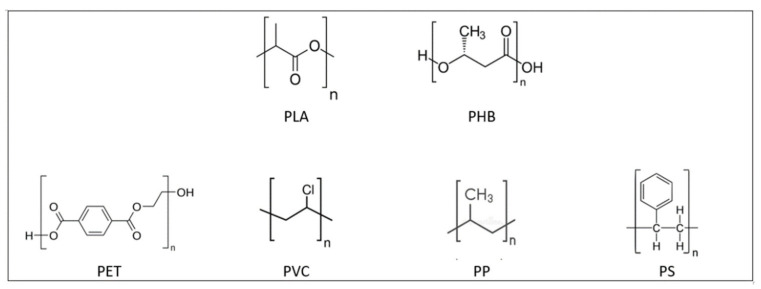
Chemical structures of biodegradable: Polylactic acid (PLA) and polyhydroxybutyrate PHB); and nonbiodegradable polymers: Polyethene terephatalate (PET), polyvinyl chloride (PVC), polypropylene (PP), polystyrene (PS).

On the other hand, PHAs gained considerable interest as a green alternative to petrochemically derived plastics, as they are biocompatible, biodegradable and synthesised from renewable resources [40,41]. PHB is the only polymer from the PHAs family to be produced in large quantities. This material is considered an aliphatic polyester with a linear polymer chain, composed of monomers of 3-hydroxybutyrate with a chromophoric carbonyl group. Being a member of the PHAs, PHB is also characterised by having a methyl (CH_3_) as an alkyl replacing group, which provides it with a hydrophobic charge. The regularity of the polymerised PHB chain has a direct influence on its degree of crystallinity that, in turn, is influenced by the synthesis route used. Isotactic PHB, which has chiral carbon in absolute configuration R, is obtained through bacterial fermentation, while syndiotactic PHB is synthesised through a synthetic route from monomers with setting R and S. As isotactic PHB presents a more regular structure, it will allow a higher crystallinity than syndiotactic [42]. Favourable PHB properties in terms of melting point, strength, modulus and barrier properties promotes it as a substitute for PP, low density polyethene (LDPE), polyvinyl chloride (PVC) and PET in packaging applications. Differences in chemical structures between PLA, PHB and previously mentioned commonly used plastics are shown in Figure 3.

Nevertheless, as a bioplastic, PHB has drawbacks, such as being brittle, hard and thermally unstable, making it challenging to use for applications like injection moulding in food industries [43,44]. As a pure material, PHB is highly crystalline (around 80%), resulting in the previously mentioned brittle nature and low elongations. The brittle nature of PHB is associated with a secondary crystallisation of the amorphous phase at ambient temperature. Another important issue is the glass temperature (T_g_) of PHB. The T_g_ is close to room temperature resulting in secondary crystallisation taking place during storage, which, combined with a low nucleation density feature, leads to large spherulite formations which can grow over long durations leading to inter-spherulitic cracks [43]. Generally, spherulites are formed when PHB is crystallised from the melt, with band spacing between them depending on the crystallisation temperature [45]. Cracks are always present within spherulites in melt-crystallised PHB, and subsequent growth of the cracks leads to failure of the polymer. Two distinct types of crack exist in PHB spherulites, which can run either radially or circumferentially within the spherulites. Radial cracks occur more frequently in films crystallised at lower temperatures, while circumferential cracks occur when PHB is crystallised at high temperatures [46]. Another problem with PHB processing is the narrow processing window. The melting temperature of PHB is around 180 °C, therefore, processing temperature should be at least 190 °C. However, thermal degradation at this point happens rapidly, drastically reducing the acceptable residence time in the processing equipment to a few minutes only [47].

However, notwithstanding the limitations of PLA and PHAs, these bioplastic polymers have the potential to be fine-tuned to extend their application range comparable to fossil-based thermoplastics.

### 3.2. Bioplastic Barrier Performance

Inadequate fluid and gas barrier properties strongly impedes the utilisation of biopolymers in applications, such as the food-packaging sector. The established utilisation of PET and polyolefin family polymers in packaging applications is due to their combination of low cost, transparency, good barrier (to oxygen and water vapours), and mechanical characteristics. The oxygen permeability of PET (0.04 barrer) is much stronger than that of the recently developed biopolymers [48]. Among previously mentioned biopolymers, the most widely used—PHB stand apart having significant gas barrier properties (0.01 barrer), which are comparable to benchmark polymers, such as PET [49]. However, PHB’s brittle mechanical nature precludes its suitability for food packaging applications. PLA, while having tunable mechanical properties, has oxygen permeability levels in the region of 0.26 barrer, restricting its use within several food-packaging applications [50]. As an approach to overcoming these barrier limitations, the addition of fillers to block the gas and moisture molecular pathways through the polymers, on a nano-micro scale level, is an attractive option [51].

An ideal filler should have a high surface area, aspect ratio and suitable chemical compatibility to provide enhanced mechanical and gas barrier properties at low filler content. The filler geometrical characteristics are an important factor in reducing gas permeability. The higher the aspect ratio, the greater the surface activity, which leads to an increase in mechanical properties, as well as gas barrier properties of the polymer matrix.

**Figure 4:**
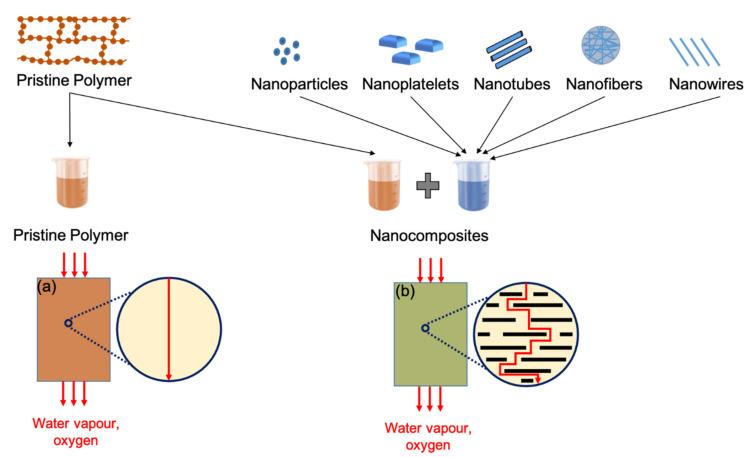
Effect of filler addition on gas barrier properties of nanocomposites: (**a**) Poor barrier properties in pristine polymer, due to direct diffusion pathways for gas molecules, (**b**) improved barrier properties in nanocomposites due to longer diffusion pathways.

In terms of the orientation of fillers inside the polymer matrix, the Nielson model is commonly used. This is an ideal case where the orientation of the filler is perpendicular to the direction of diffusion and is generally not readily achievable. The modified version of the Nielson model is proposed by Bharadwaj by introducing the orientation parameter (S) [52]. If S = 0, designates perfect orientation and the Bharadwaj model will be reduced to the Nielson model, and maximum permeability reduction will be observed as indicated in Figure 4. Different models based on volume fraction and aspect ratio have also been developed to compare theoretical data with experimentally investigated gas barrier results [53].

Another aspect to be considered while studying the bioplastics’ barrier properties is the dispersion and interchain compatibility of fillers within the bioplastics’ matrices. Well exfoliated nano-fillers in polymer matrices give optimal reinforcement and contribute to other material performance characteristics [51]. A major issue with the dispersion of fillers in polymers is their hydrophilic nature, which causes inefficient compatibility with the hydrophobic polymer phase. Therefore, treatments are adopted to promote better interactions and good dispersion between the polymer phase and the fillers [54]. Moreover, the structural characteristics of the filler define the contribution imparted to the mechanical and gas barrier properties of polymers. The structural format of fillers dramatically impacts the gas barrier properties of their host polymers [55]. This is due to the higher crystallinity, which increases the effective path of diffusion and impedes the passage of gas molecules through the polymers rendering them suitable for packaging applications.

**Figure 5:**
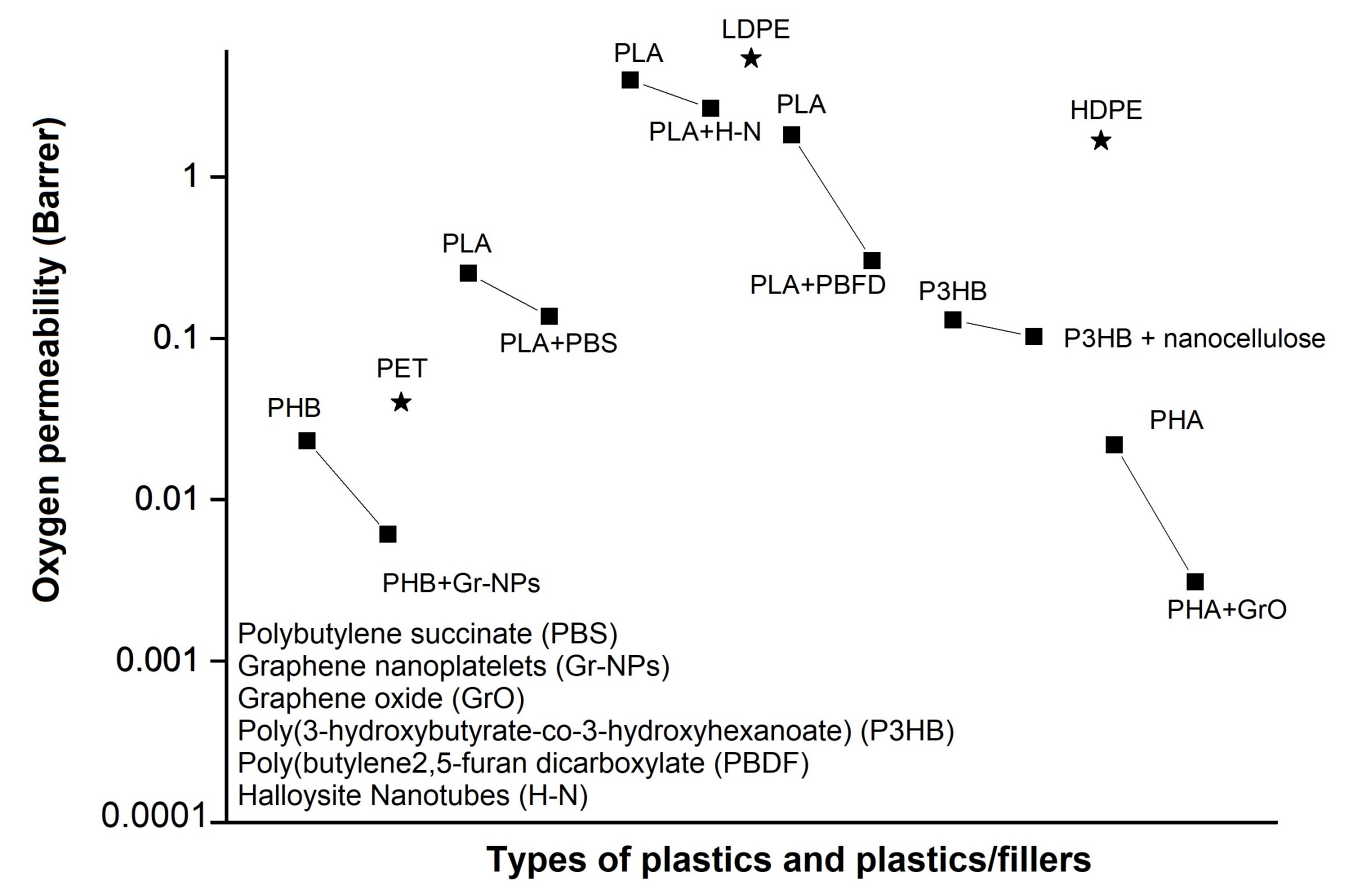
Effect of nanofillers on oxygen permeability of various biopolymers.

Figure 5 demonstrates the effect of fillers addition on the barrier properties, especially the oxygen (O_2_) permeability of commonly used petroleum-based plastics and biopolymers. The data is compiled by converting oxygen permeability values from different units into a single unit (barrer). The addition of smaller amounts of fillers in biopolymers has drastically reduced the permeability of oxygen, fulfilling the criteria of ideal gas barrier material (LDPE, PET, and high density polyethene (HDPE)). Among biopolymers, PHAs showed more hindrance to the passage of gas molecules in their pristine polymers as compared to mentioned petroleum-based polymers. Functionalised graphene oxide (Gr-O) proved to be the best filler. The impressive reduction of oxygen permeability by Gr-O could be related to the strong interfacial adhesion between Gr-O and PHA polymer matrix [56].

### 3.3. Bioplastic Processing and Formulation

Blending and composite formation is an established route for achieving improved technical and processing performance within polymer engineering. Blending PHAs, in particular, PHB with other polymers, offers opportunities to improve processability by lowering the processing temperature and reducing the brittle nature of these biopolymers. The physical, chemical and molecular architectural aspects dictate the enhancement of the polymer blend achievable through compounding techniques and introduced additives. Plasticiser additives can improve polymer viscosity and improve chain mobility during processing. Thermal stabilising additives can be used to eliminate premature degradation of polymers during processing, such as antioxidants, which guard against the presence of oxygen in the processing environment. Compatibiliser additives can improve miscibility between polymers by inducing flexible physical dipole-dipole interactions, or hydrogen bonding [57]. Nanocomposite additives, such as nanocrystals and nanofibres, can significantly improve the mechanical strength and gas barrier properties of the polymers if they are well dispersed in the biopolymer matrices. Natural fibres as an example of nanocomposite have been recently introduced as the main component in fibre reinforced biopolymer composites [58]. The intermolecular hydrogen bonds connecting the polymer chains of natural fibres provide a linear crystalline structure with a tensile strength reaching 15 GPa [59]. Such great strength is also accompanied by other advantages, as low cost, abundance, biodegradability, easy recyclability and fabrication of low weight composite materials [58]. All these properties made natural fibres perfect candidates as fillers in biopolymer composites and can compete with glass or carbon fibres. Accordingly, several studies were performed to evaluate the effect of incorporating natural fibres in biopolymer composites to improve the composite’s mechanical and barrier properties. Among natural fibres, cellulose [60,61,62,63,64,65,66], hemp [67,68,69,70,71,72,73], kenaf [74,75,76,77,78] and flax [79,80,81,82,83,84,85,86,87,88] were the most studied ones. It is worth mentioning that nanocomposites of natural fibres or crystals added as fillers without plasticiser or compatibiliser, results in their poor dispersion and decrease the quality of polymer composite. Alternatively, plasticisers of hydrophilic nature, when mixed with biopolymers or their blends, tend to increase the wettability and O_2_ permeability and deteriorate the barrier properties of the polymer composite.

Thus, to obtain better performance of nanocomposites with plasticisers or compatibilisers in biopolymer composites, both additives should be used together. The addition of nanocomposites with plasticisers improves the interfacial adhesion between the nanocomposites and the polymer matrix, allowing better dispersion and consequently provides a more tortuous path for gas and water and increase the barrier properties. Other approaches were introduced to improve the dispersion of the nanocomposites of natural fibres or crystals in polymer matrices. These include physical and chemical treatments of the nanocomposites before mixing with biopolymers. Bio-based coatings were also applied to natural fibres reinforced biocomposites as a means of inducing the hydrophobicity, and thus, improve the barrier properties of the biocomposite.

#### 3.3.1. Blends and Composites

**Figure 6:**
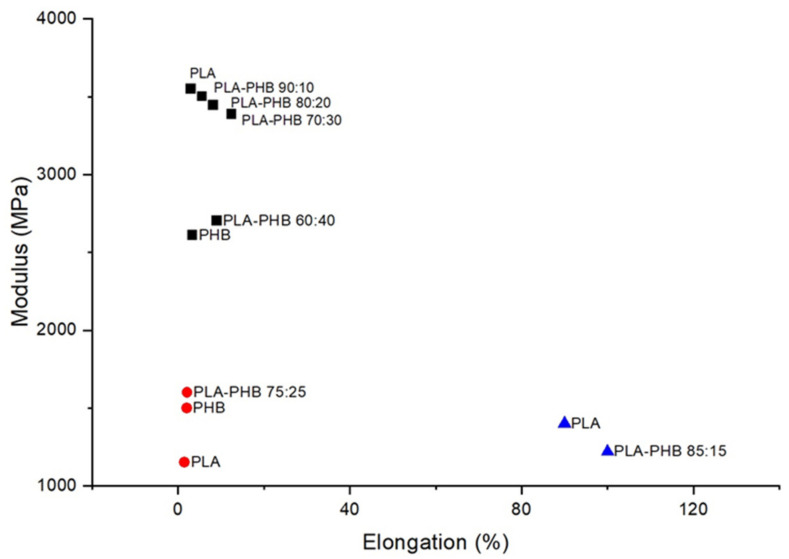
Young’s Modulus (MPa) vs. elongation at the break (%) of PLA, PHB and their blends, reported by Jandas et al. (black squares), Data from Ref. [57], Armentano et al. (blue triangles), Data from Ref. [91] and Arrieta et al. (red circles), Data from Ref. [94].

As previously mentioned, the processability and formability of PHB represent a drawback in industrial applications; blending with PLA provides a potential route to facilitating its introduction in the market, while also improving PLA properties at the same time. Several studies on PLA-PHB blends have been conducted in recent years, with the results typically showing a slightly higher Young’s modulus than neat PHB and neat PLA [89]. Blends with PHB content of 50% or higher have shown lower values of tensile stress and elongation at the break in comparison to pure PLA [90]. However, above 60% PLA content has been reported to increase elongation at the break by up to 12%, with values even comparable to typical thermoplastics achieved on the addition of plasticisers [57,89,91,92,93]. Furthermore, the PLA-PHB 75:25 blend demonstrated higher mechanical performance than neat PLA, and greater impact resistance than the homopolymers on their own [89,94]. Jandas et al. reported the incorporation of PHB within PLA matrix in different ratios resulting in intermediate properties for the blends. The ductility of PLA increased consistently as PHB concentrations increased from 10 to 30 wt%. The maximum increase in percentage elongation was observed in the 70:30 ratio, suggesting some degree of molecular interaction between the macromolecules of PLA and PHB within the blend (Figure 6). However, tensile modulus and tensile strength were considerably decreased in the case of the blends, compared to pure PLA, as well as a corresponding decrease in stiffness. Blends prepared at 70:30 ratio were used for trial with compatibilisers and preparation of blend composites, due to the optimum elongation at the break and impact strength exhibited [57].

#### 3.3.2. Compatibilisers and Plasticisers

Facilitation of processability and improved flexibility in PLA/PHB blends is achievable using plasticisers [94]. Plasticisers are available as cost-effective, readily available materials on the market and are also generally of natural origin and include: Oxypropylated glycerin (or laprol), glycerol, glycerol triacetate, 4-nonylphenol, 4,40-dihydroxydiphenylmethane, acetyl tributyl citrate, salicylic ester, acetylsalicylic acid ester, soybean oil, epoxidised soybean oil, dibutyl phthalate, triethyl citrate, dioctyl phthalate, dioctyl sebacate, acetyl tributyl citrate, di-2-ethylhexylphthalate, tri(ethylene glycol)-bis(2-ethylhexanoate), triacetine, and fatty alcohols with or without glycerol fatty esters. Blends of PEG (2–5%) and PHB produced by solvent casting technique has been demonstrated to increase the elongation at the break by up to about four times compared with the original neat PHB. This behaviour was attributed to a plasticising effect of PEG, which acts to weaken the intermolecular forces between the adjacent polymer chains. The changes in free volume reduced the melting temperatures of the system and are also associated with an accompanying reduction in tensile strength [95].

**Figure 7:**
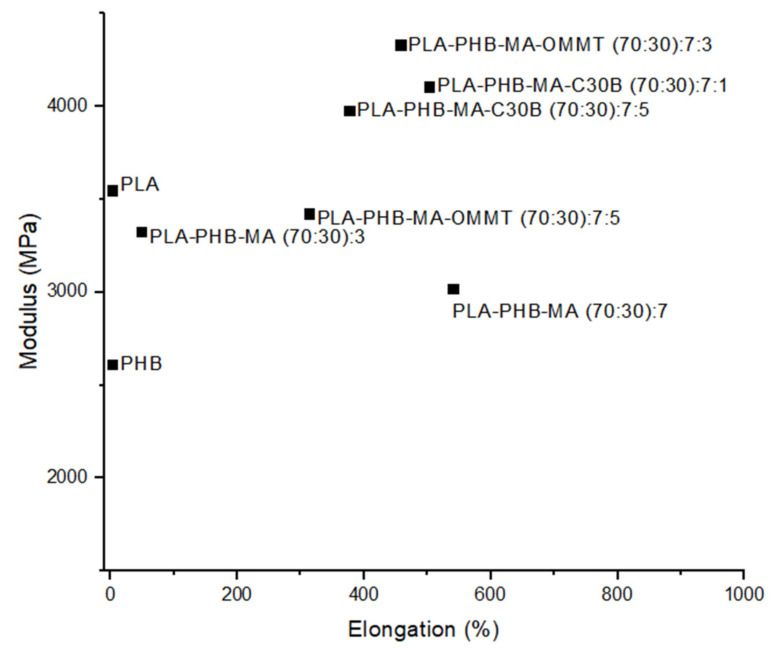
Young’s Modulus (MPa) vs. Elongation (%) of PLA, PHB and their blends using MA as a compatibiliser, together with OMMT and C30B nanoclays, Data from Ref. [57].

As shown in Figure 7, compatibilisers, such as maleic anhydride, have been applied to PLA and PLA/PHB blends to impart additional flexibility and improve the blend’s young’s modulus by the induction of flexible physical interactions, including dipole-dipole or hydrogen bonding [57]. For industrial purposes, considerable attention needs to be paid to the selection of suitable plasticisers or compatibilisers as most tend to negatively impact other mechanical properties, such as lowering barrier properties of bioplastic blends, which can restrict their use in packaging applications [89].

#### 3.3.3. Natural Fillers

The use of naturally sourced fibre-based fillers, with their considerable tensile strength and high sustainability, presents a key route to facilitating polymer circularity. The hydrophilic nature of bare natural fibres with no surface treatment requires an address, due to the incompatibility with hydrophobic biopolymer. Biopolymer composites reinforced with untreated natural fibres typically exhibit non-uniform fibre dispersion with interfaces promoting crack formation. These material defects can lead to premature mechanical failure of the composites [58,59,96]. These defects are attributable to the natural fibre hydrophilicity, which hinders proper mixing with the biopolymer hydrophobic matrix causing poor fibre/matrix interfacial bonding [59]. While high susceptibility of moisture absorption by the hydrophilic natural fibres can support the growth of fungi and bacteria and deteriorate the physical and mechanical properties of the bio-composites [58], this fact can be a highly useful feature that can be availed of post use for achieving biocyclability.

Different approaches are available to enhance the interfacial adhesion between the fibres and matrix, resulting in the fabrication of biocomposites with better mechanical and barrier properties. Among these solutions is the surface modification of natural fibres. Surface modification can result in increasing the sites of reaction, offering new functionality to the fibre surface and enhancing surface roughness by removing impurities [59]. Such modification will lead eventually to the improvement in mechanical properties and reduction of the water absorption of the fabricated biocomposite. The techniques applied to modify the natural surface fibres before inclusion in biopolymer matrices include physical, chemical, biological treatments and their combinations. Some examples of physical treatment techniques are calendaring, stretching, hybrid yarns production and thermal treatments; while chemical techniques include, alkali swelling, silane modifications, graft copolymerisation, and treatment with isocyanate, mercerisation [97].

A representative selection of the recently applied surface modifications for natural fibres reinforced biocomposites are given in Table 3.

Combinations of natural materials with petroleum-based plastics is an option that is also under development. While facilitating the high mechanical performance, considerations are required when using this approach as further dilemma’s may be posed regarding factors, such as continued resource depletion dependencies and degradation and biodegradation pathways, which potentially lead to increased microplastics production.

#### 3.3.4. Bio-Coatings

The application of bio-based coatings to biocomposites and natural fibre reinforced biocomposites is a promising approach proposed to overcome the significant water uptake propensities of natural fibres and increase the moisture resistance of bioplastics for fluid barrier property application requirements. Exposure to long term environmental/hygroscopic ageing necessitates the induction of a higher level of hydrophobicity in chemically modified natural fibre reinforced bio-composites and bioplastics in general. Introducing bio-based coatings to natural fibres reinforced biocomposites, ensures the environmentally friendly and biodegradable nature of fibres. Besides, bio-based coatings are obtained from renewable resources and have superior hydrophobic characteristics [62]. For instance, polyurethane (PU) coatings were first introduced as bio-based coating resins in the 1950s [98]. PU coatings mainly provide their composites with high solvent resistance, hydrolytic stability, resistance to acid–base conditions and weather-ability [62]. Currently, most of the industrially produced PUs are petroleum-based polyols. Thus, renewable resources, such as vegetable oil [99,100,101], canola oil [102], soybean oil [103,104,105] and castor oil [106,107,108], are thoroughly investigated and were able to produce PU coating of competing properties to that of petroleum-based ones.

Polyfurfuryl alcohol (PFA) is another attractive type of bio-based coatings that can be used for barrier property application requirements. PFA has a low cost manufacturing process and can be obtained from natural resources as the agricultural residue of wheat, birch wood, hazelnut shells, corn, rice hulls, oat and sugar cane [62]. In addition, PFA’s hydrophobicity, great heat distortion temperature and resistance to chemical erosion make it an excellent candidate as a coating material for bioplastics and natural fibres reinforced biocomposites.

Despite such potentials of PU and PFA as bio-based coatings, there is almost no work presented in the literature on the application of PU and PFA as coatings for natural fibre reinforced composites or biocomposites, except for a recent study done by Mokhothu et al. [109]. This study proposed using PU and PFA as bio-based coatings to composites containing flame-retardant treated natural fibres (flax) and phenolic resin. For three days, uncoated and coated samples were subjected to 90 °C and 90% relative humidity. Analysis was performed to the relative moisture content and mechanical properties and compared with the commercially available water-resistant product (FIRESHELL^®^ (F1E)). Concerning the mechanical properties, PFA coated samples showed the highest modulus value (1.93 GPa) after being subjected to environmental conditioning with respect to uncoated (1.59 GPa); PU (1.05 GPa) and F1E (0.98 GPa) coated composites. Besides, the PFA and PU coated samples showed high stress at the break and a decreased elongation at the break in comparison to F1E coated ones. The moisture content of the conditioned PFA and PU coated composites was significantly reduced by 75% and 30%, respectively, when compared to uncoated and F1E coated composites [109].

## 4. Bioplastics Biodegradability

There is an important distinction between degradable polymers and biodegradable polymers. Degradable polymers are defined as polymers that can be depolymerised or recycled under controlled conditions and processes. According to the American Society for Testing and Materials (ASTM) definition, biodegradable polymers are polymers that can undergo decomposition into carbon dioxide, methane, water, inorganic compounds, or biomass, which can be measured by standardised tests, in a specified period, reflecting available disposal conditions (ASTM standard D6813). The mechanism of biodegradation is that the molecular weight of biodegradable polymers reduced, due to hydrolysis and oxidation, followed by breaking down into natural elements, such as water and carbon dioxide, via microorganisms. Aliphatic polyesters are the most economically viable biodegradable polymers, with PHB being among the most mechanically promising. The structural changes to the polymer molecules can be described in terms of three main categories of actions or mechanisms, namely, chain depolymerisation, random chain scission, and substituent reactions [110]. As defined by the IUPAC, depolymerisation is the process of converting a polymer into a monomer or a mixture of monomers/oligomers. Therefore, chain depolymerisation means the chain reaction is responding to the transformation of the macromolecular polymer chain into its constituent micromolecular monomers. Random scission is defined in the IUPAC Gold Book as a chemical reaction resulting in the breaking of skeletal bonds. It is also defined as a degradation mechanism that assumes a random cleavage of bonds along the macromolecular polymer chains [111]. This leads to the production of fragments that steadily decrease in length, which may eventually be small enough to allow for the removal of micromolecules. Substituent reactions refer to the kinetic reactions carried out by the constituent monomers of a polymer chain, which differ between polymers. According to Ghosh (1990), each kind of substituent has a characteristic chemical nature and reactivity [112]. However, as substituent reactions can only be observed at relatively low temperatures, substituent reactions only assume prominence when initiated and accomplished at temperatures lower than those of the breaking temperature of main chain bonds of a polymer.

Several factors affect the degradability of a polymer. In general, the surface conditions, the first-order structures, and the high order structures of a polymer play a major role in determining the rate of degradation. Surface conditions, such as hydrophilicity and surface area, directly correspond with the overall degradability of a polymer. Additionally, external environmental factors, such as humidity and temperature, also affect the overall degradability. Humidity introduces water molecules to a polymer and may result in a hydrolysis process, depending on the susceptibility or hydrophobicity of the polymer. Furthermore, the crystallinity of a polymer is also proportional to the degradability of a polymer, so that the lower the degree of crystallinity, the higher the degradability of the polymer. According to Tokiwa et al. (2009), this can be attributed to the fact that enzymes generally interact with the amorphous regions within a polymer, which are loosely packed together as compared to the crystalline regions. Moreover, the melting temperature (*T_m_*) of polyesters greatly affects their enzymatic degradation. This is evident from the fact that aliphatic polyesters and polycarbonates with low Tm have a greater biodegradability than aliphatic polyurethanes and polyamides, which have higher Tm [113]. This is due to the large melting enthalpy change values of the latter, which can be attributed to the presence of hydrogen bonds among the polymer chains. The introduction of heat into a polymer matrix generally weakens the intermolecular bonds, resulting in an increased rate of degradation. With biodegradation specifically, the microbial species introduced to the polymer, directly correlates with the level of microbial activity, which in turn determines the rate of degradation of a biodegradable polymer [114]. The degree of microbial activity is also heavily influenced by nutrient and oxygen content in the biodegradation environment.

As described earlier, selected microorganisms can produce and storing PHAs. The ability to synthesise these molecules does not imply the capacity to also degrade them, in the case where extracellular hydrolases capable of converting polymers are also expressed [115,116]. Under nutrient-limited conditions, degradation occurs when the limitation is removed. Currently, six hundred PHA depolymerases from various microorganisms have been identified and categorised within eight families [117]. The degradation of these polymers is affected by many factors, such as type of enzyme, temperature, moisture, and nutrients composition [118]. Degradation rates of PHAs are also related to the microbial population density. It was shown that during degradation of P(3HB-co-3HV) copolymer, microbes at first attach to the polymer and then begin secreting degrading enzymes. Although PHA producing/degrading microbes usually express high specificity towards P(3HB), many microbes have been identified with wide substrate specificity. *Xanthomonas* spp., for example, has the ability to produce enzymes for PHAs with aromatic side chain degradation and can also degrade P(3HB), P(3HO), and poly-3-hydroxy-5-phenylvalerate (P(3HPV)) [119]. The type of polymer also plays an important role in degradability. In addition to the presence of side chains, length and composition are also significant factors. Manna et al. report that homopolymers have higher degradation rates in comparison with copolymers of PHAs [120]. Other studies have demonstrated opposing results [118], which may be explained because, in these cases, the experiments were conducted in natural environments where previously mentioned factors (nutrients, moisture, temperature etc.) were non-controllable. Kusaka et al. showed that PHAs degradation ability is negatively correlated to the *M_w_* and crystallinity [121]. The format and shape of the polymer material is also a significant factor for PHAs degradation, with thin films degrading faster than thicker films. Soil and climatic conditions are further factors that can affect the PHA degradation rate [28,118]. Boyandin et al. examined PHA films degradation response and reported that humid and the hot Vietnamese climate facilitated degradation of PHA [122].

**Figure 8:**
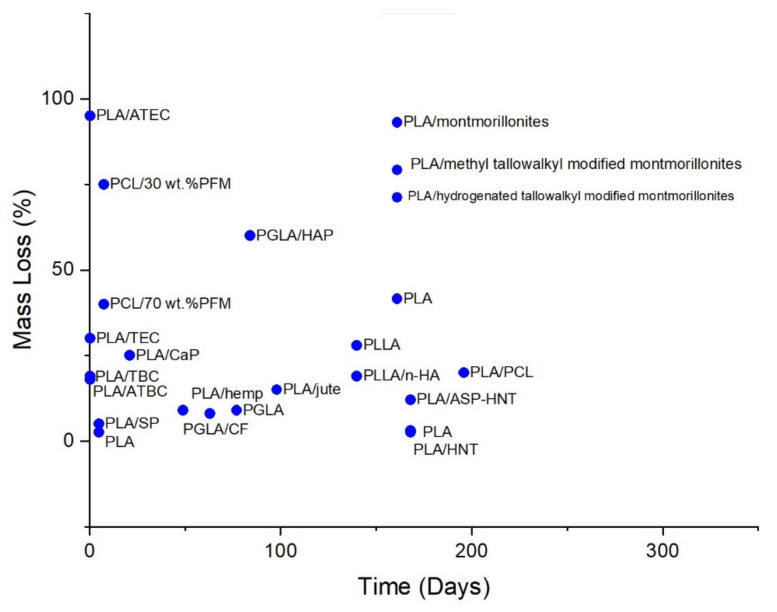
Mass Loss percentage of composites in various hydrolytic degradation environments.

**Figure 9:**
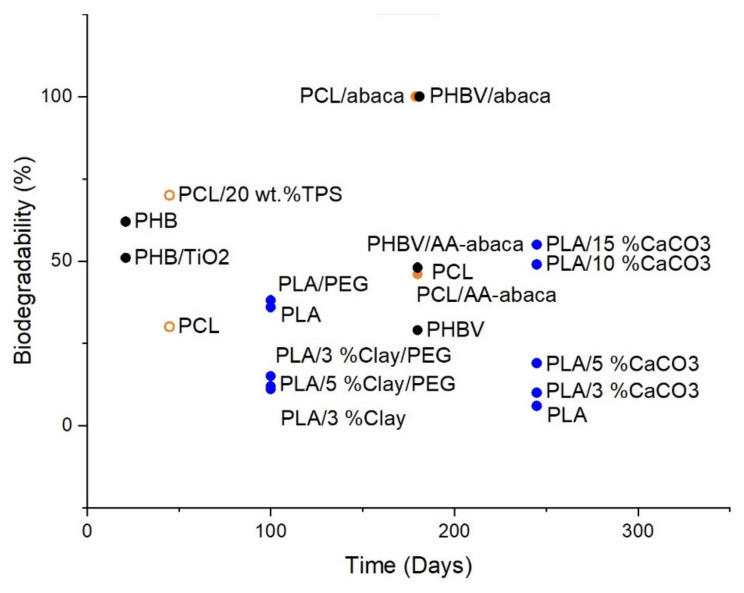
Biodegradability of PLA, PCL and PHB with the addition of various fillers and plasticisers in various soil burial degradation environments.

Additives, such as fillers, are another factor that can affect the biodegradability of the bioplastic in which they are added, as demonstrated in Figure 8 and Figure 9. There is no general guarantee that the addition of fillers will enhance or inhibit biodegradability as the effect of fillers on a polymer is mainly dependent on its chemical and physical aspects, such as size, geometry, surface area, and the surface energy of its particles [123] (Murphy, 2001). These aspects directly affect the overall degradation ability of a polymer. In general, the effect of additives on the biodegradability of a polymer is largely dependent on the properties of the additives, such as hydrophobicity and amenability to bacterial growth on the surface. 

Aframehr et al. report a study on the effect of calcium carbonate (CaCO_3_) in soil burial biodegradation where the CaCO_3_ fillers, act to increase the biodegradability of PLA. The weight loss of CaCO_3_ nanocomposites is approximately two times higher than other nanocomposites, with a weight loss of around 55% for PLA/15% CaCO_3_, 49% for PLA/10% CaCO_3_, 19% for PLA/5% CaCO_3_, 10% for PLA/3% CaCO_3_, and around 6% for neat PLA after a soil exposure time of 35 weeks [124]. A study by Teramoto et al. investigated the effect of treated and untreated abaca fibre filler on the biodegradability of poly(3-hydroxybutyrate-co-3-hydroxyvalerate) (PHBV) after being subjected to a soil-burial environment for a duration of 180 days. Neat PHBV exhibited the least biodegradability at only around 29% weight loss, followed by PHBV/AA-abaca at around 48% weight loss, and lastly, PHBV/untreated abaca with the highest biodegradability, which can be seen in the high degree of fragmentation after 60 days [125].

Altaee et al. conducted a study on the biodegradation of PHB and titanium oxide (PHB-TiO_2_) composites in a soil burial environment with pH 7.30 and a humidity of 80% at 30 °C and found that PHB-TiO_2_ exhibits a lower weight loss of only ~51% after three weeks as compared to ~62% weight loss of neat PHB through the same duration [126]. Paul et al. (2005) studied the degradation of nanocomposites of PLA with unmodified and organo-modified montmorillonites and found that montmorillonites filler enhances hydrolytic degradation. PLA with unmodified montmorillonites exhibited the greatest decrease in *M_w_* after 23 weeks of hydrolytic degradation with a 93.1% loss in *M_w_*. These results were followed by PLA with montmorillonites treated with bis-(2-hydroxyethyl) methyl tallowalkyl ammonium cations and PLA with montmorillonites treated with dimethyl-2-ethylhexyl (hydrogenated tallowalkyl) ammonium cations at 79.2% and 71.2% *M_w_* loss, respectively. In comparison, unfilled PLA is found to only have a 41.6% *M_w_* decrease compared to its initial value [127]. Chen et al. found that PLA with halloysite nanotubes (HNTs) as filler has a greater rate of hydrolytic degradation as compared to neat PLA as shown from the mass reduction of 3.1% for PLA/HNT as compared to that of neat PLA at only 2.6% in an in vitro environment in SBF at 37 °C by the end of the 24th week of degradation. It was also reported that PLA with HNTs surface treated with 3-aminopropyltriethoxysilane (ASP) has an even greater hydrolytic degradation, due to better interfacial adhesion between PLA and HNTs, which is evident from the mass reduction of 12.1% [128]. A study by Navarro et al. (2005) investigated the effect of the addition of calcium phosphate (CaP) glass to PLA on its hydrolytic degradability. During the first three weeks of the degradation, PLA/CaP composites experienced a greater weight loss than neat PLA, but an increase of weight of the PLA/CaP composite was reported after three weeks. This may be credited to forming hydrated calcium phosphate precipitate on the composite. The maximum weight loss exhibited by week 3 is 25%, and a final weight loss percentage of about 22% on week 6. In a comparison, the weight loss of neat PLA is around 1% from week 3 through week 5 [129]. Moreover, a study by Huang et al. (2013) investigated the hydrolytic degradability of poly(L-lactic acid) (PLLA)/nanohydroxyapatite (n-HA) and found that the rate of degradation of PLLA/n-HA composite was slower than neat PLLA. This is evident from the weight loss of only around 19% for PLLA/n-HA composite and about 28% for neat PLLA after 20 weeks in a PBS environment with an initial pH of around 7.4 [130]. A study by Valapa et al. (2016) investigated the hydrolytic degradation behaviour of sucrose palmitate (SP) reinforced PLA (PLA-SP) nanocomposites in acidic (pH 2), basic (pH 12), and neutral (pH 7) hydrolytic degradation environments and found that the rate of degradation is increased with the addition of sucrose palmitate. This can be seen in the mass loss percentage of about 5.1% for PLA-SP as compared to only around 2.6% mass loss percentage of neat PLA in a pH 7 degradation solution at 35 °C after 115 h [131].

Alternatively, the alterations proposed by the addition of plasticisers and compatibilisers to bioplastics directly affect their degradability since plasticisers and compatibilisers decrease the glass transition temperature (*T_g_*) of the polymers they are blended with, as shown in Figure 8 and Figure 9 [132]. Like fillers, there is no general guarantee that the addition of plasticisers will positively or negatively impact biodegradation as it is highly dependent on the properties of the plasticisers used. Some of the more common forms of plasticisers include citrate esters and phthalates (phthalate, isophthalate, terephthalate), the latter being biodegradable as degradation by microorganisms is considered as the most effective method of degradation for phthalates plasticisers [133]. A study by Labrecque et al. (1997) researched using triethyl citrate (TEC), tributyl citrate (TBC), acetyl triethyl citrate (ATEC) and acetyl tributyl citrate (ATBC) as plasticisers for PLA and their effect on enzyme-catalysed hydrolytic degradation. The study found that all citrate esters enhanced the degradability of PLA. At a concentration of 20%, ATEC plasticised PLA has the highest weight loss of around 95%, followed by neat PLA at around 48%, and a decreased rate of degradation in TEC, TBC, and ATBC with weight losses of around 30%, 19%, and 18%, respectively, after a degradation period of 6 h [134]. A 2009 study by Ozkoc and Kemaloglu found that the addition of PEG and clay plasticisers to PLA decreases the rate of biodegradation after exposure to the composting environment for 100 days. This is evident from the weight loss percentage values of about 15%, 12%, and 11% for PLA/3%Clay/PEG, PLA/5%Clay/PEG, and PLA/3%Clay, respectively, in comparison to that of neat PLA with a 36% weight loss. However, the weight loss percentage of PLA/PEG shows a slight increase as compared to neat PLA with a value of around 38% weight loss [135].

Careful selection and monitoring of additives and composite formation, hence, has the potential for exploitation both to enhance the target mechanical properties and simultaneously promote biodegradation.

## 5. Conclusions

The fundamental many-faceted aspects of biopolymer architectures afford greater versatility for configuration and exploitation compared with the main ubiquitous recalcitrant synthetic petroleum-based plastics. This is a pivotal trait that is available for harnessing to enable bioplastics to meet both the high mechanical and barrier performances of their petroleum-based counterparts together with full sustainable biodegradability and circularity. Their very bio-nature means bioplastics are inherently more elaborate at a structural level. This increases accessibility to bio-interactions for enzymatic biodegradation, biodepolymerisation and biorepolymerisation, as well as supporting routes to improving mechanical and barrier performances. While several bioplastics have already achieved certain mechanical and barrier performance criteria, which are equivalent and even exceed those of corresponding petroleum-based plastics, courses of action for resolving the remaining limitations are becoming increasingly accessible.

Here, avenues for the advancement of the performance of bioplastics with respect to mechanical and barrier properties alongside biodegradability are discussed. The key architectural features properties are *M_w_* and crystallinity, which typically exhibit an inversely dependent relationship with mechanical performance and biodegradability for both bioplastics and petroleum-based plastics. Increasing *M_w_* and crystallinity are generally associated with higher mechanical performance and decreased biodegradability as lower crystallinity corresponds to looser chain packing, facilitating enzyme access.

Both macro and micro strategies have the potential for dual positive correlation on the mechanical and biodegradability performances of bioplastics. Regarding the macro approach, new possibilities are afforded, such as harnessing intricate bioplastic structures and formats, which include microfibrillar frameworks and combination with advanced methods, such as in situ polymerisation. Blending and compounding with additives as selected fillers, plastisisers and compatibilisers are also being demonstrated for improved mechanical features, without decreasing biodegradability.

Whereas, regarding the micro approach, expanding PHAs families and large numbers for possible monomer units, present the potential to engineer biodegradable plastics with equivalent target petroleum plastic performances without associated environmental pollution. Further routes include metabolic pathway alteration, design of high specificity substrates, intricate copolymer and block copolymer and genetic modification to produce strains to achieve next-generation multifunctional biopolymers.

In these respects, bioplastic polymers, in contrast to Petroleum-based plastics, have not yet been tailored or even adequately explored to establish their capacities for current and future applications. Given the range and diversity of options available for bioplastics development, there are excellent prospects to extend their application range on a comparable scale to fossil-based thermoplastics and beyond. Further innovations can be expected as the knowledge and new capacities for the manipulation of biopolymers advances, and spawns outputs in related and novice disciplines. The realisation of high performance plastics, without recalcitrance, pollution or resource depletion and switching to regenerative low carbon circularity, has the potential to both safeguard and promote future prosperity for the planet and its inhabitants.

## Figures and Tables

**Table 1:** Chemical structures of monomers described as units of PHA copolymer producing strains.

3-Hidroxyacids	Structure
butyric (3HB)	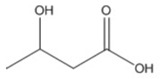
hexanoic (3HHx)	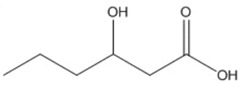
octanoic (3HO)	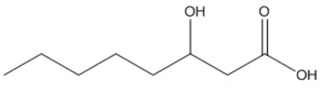
decanoic (3HD)	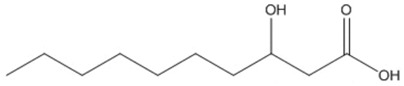
dodecanoic (3HDD)	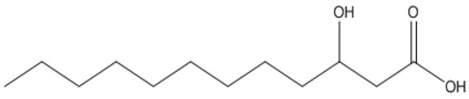

**Table 2:** Chemical structures of polymers commonly found as building blocks in PHA related block copolymers.

Polymer	Structure
poly(6-hydroxyhexanoate)	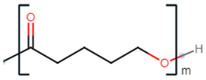
poly(3-hydroxyoctanoate)	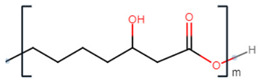
monomethoxy-terminated poly(ethylene glycol) (mPEG)	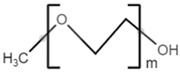
poly(ethylene glycol)	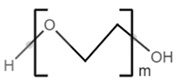

**Table 3:** Surface treatment of natural fibres reinforced bio-composites.

Process	Natural Fibre Used	Biopolymer Matrix	Outcomes			Ref.
			Interfacial Adhesion	Mechanical Properties	Barrier Properties	
Treatment with compatibilisers; Polyglycerol polyglycidyl ether (SR-4GL), Trimethylol propane polyglycidyl ether (SR-TMP), and (Polyglycerol polypropyleneoxide (SC-P1000)	Cellulose fibres	PLA	Improved interfacial adhesion between fibres and PLA and		Inhibited degradation of the PLA matrix	[65]
STEFAC TM 8170, surfactant modification	Cellulose fibres	PLA/PHB		Enhanced mechanical performance	Improved water resistance, reduced oxygen and UV-light transmission, as well as appropriate disintegration in compost	[94]
Alkali treatment	Kenaf fibre	PHB		Reduction in the crystallinity of PHB (up to 6% reduction), making it more ductile, and improvement of the flexural modulus by up to 11%.		[75]
Silane treatment	Flax fibre	PLA	Improvement to fibre/matrix adhesion with 2% *w/w* silane content, yet further improvement of the fibre-matrix interface can be partially resolved by silane/alkali treatment combination.	Improved mechanical properties		[80]
Alkali treatment	Flax fibres	PLA		*T_g_* values of fabricated bio-composites were lowered by 10 °C for 10% NaOH treatment and 15 °C for 30% NaOH treatment		[82]
Treatment with ethylene plasma	Flax fibres	PHB	Improved interfacial adhesion strength in the bio-composite		Improved thermal resistance	[88]

## Data Availability

The data presented in this study are available on request from the corresponding author.
